# Computing the T-matrix of a scattering object with multiple plane wave illuminations

**DOI:** 10.3762/bjnano.8.66

**Published:** 2017-03-14

**Authors:** Martin Fruhnert, Ivan Fernandez-Corbaton, Vassilios Yannopapas, Carsten Rockstuhl

**Affiliations:** 1 Institute of Theoretical Solid State Physics, Karlsruhe Institute of Technology, Wolfgang-Gaede-Strasse 1, 76131 Karlsruhe, Germany; 2 Institute of Nanotechnology, Karlsruhe Institute of Technology, P.O. Box 3640, 76021 Karlsruhe, Germany; 3 Department of Physics, National Technical University of Athens, Heroon Polytechniou 9, 15780 Zografou, Greece

**Keywords:** metamaterials, nanooptics, numerics, scattering, T-matrix

## Abstract

Given an arbitrarily complicated object, it is often difficult to say immediately how it interacts with a specific illumination. Optically small objects, e.g., spheres, can often be modeled as electric dipoles, but which multipole moments are excited for larger particles possessing a much more complicated shape? The T-matrix answers this question, as it contains the entire information about how an object interacts with any electromagnetic illumination. Moreover, a multitude of interesting properties can be derived from the T-matrix such as the scattering cross section for a specific illumination and information about symmetries of the object. Here, we present a method to calculate the T-matrix of an arbitrary object numerically, solely by illuminating it with multiple plane waves and analyzing the scattered fields. Calculating these fields is readily done by widely available tools. The finite element method is particularly advantageous, because it is fast and efficient. We demonstrate the T-matrix calculation at four examples of relevant optical nanostructures currently at the focus of research interest. We show the advantages of the method to obtain useful information, which is hard to access when relying solely on full wave solvers.

## Introduction

Recent advances in nanofabrication technology made the creation of large volumes of particles with complicated geometries possible [1–6]. The latter constitute the base for nanomaterials with advanced properties [7]. This also triggered the need for efficient computational tools to back up experimental findings with simulations that allow to understand the underlying principles that cause the properties of the respective nanomaterials [8–10]. With current possibilities it is fairly straightforward to calculate the scattered fields of an arbitrary object illuminated with a plane wave or more complicated illumination scenarios. They all rely on the numerical solution of Maxwell’s equations while considering a given distribution of material in space. Examples for such numerical routines are finite element solvers [11–13], finite-difference time-domain methods [14–16], discrete dipole approximation [17–18], or similar tools. This yields valuable information about the single structure. On the other hand, there are efficient methods to calculate the scattering of large clusters of spherical particles, for example the extended Mie theory [19–20]. This consideration of many particles is the prerequisite to study the emergence of properties if many of these particles are brought together to form artificial nanomaterials.

The principles of these two distinct approaches can be combined to the T-matrix method [21–23]. Using this method, the T-matrix, which contains the information about how an isolated object scatters the electromagnetic field, is calculated and subsequently used in a multiple-scattering algorithm, similar to the multi-Mie Method [20,24], to calculate the scattered fields of a large cluster of arbitrary particles.

There are several analytical and semi-analytical ways to calculate the T-matrix of simple particles with high symmetries [21,25–27]. Here, we present a method to calculate the T-matrix of an arbitrarily complex particle. Our approach only requires the object to be illuminated in a sequence of simulations with multiple plane waves. From these simulations the scattering coefficients and, ultimately, the T-matrix, are deduced from the scattered fields. These fields can be calculated with established methods, such as the finite element method but, in general, any other of the aforementioned methods can be considered as well. The resulting T-matrix is then independent of the form and direction of the external illumination. It only depends on the material and geometry of the object. The scattering coefficients that can then be calculated for any arbitrary illumination are vital for the calculation of all further quantities, such as the scattered fields and the scattering cross section for a specific illumination. The availability of the T-matrix simplifies particularly the analysis of quantities that require the evaluation of the same response of the object to a larger number of different illumination fields. A referential example therefore is the calculation of the force map for a complicated illumination in space exerted on the particle. There, the response of the same particle to the field at each spatial location of the illumination needs to be calculated. This can be a tedious task if every time a new full wave solution to Maxwell’s equations is required. The situation is much more simplified if the T-matrix of the object is known. The coefficients can also be used to determine the strength of the individual multipole contributions to the scattering for example to check if the dipole approximation is applicable. This is particularly useful in the context of the homogenization, i.e., the assignment of effective material parameters to a medium. We emphasize the fact that when only the optical response of a given particle to a given illumination is needed, it does not make much sense to calculate the T-matrix first, as it requires the solution of multiple full-wave problems. However, whenever more complicated objects are of interest, or if the response of a given structure to many different illuminations is needed, as it is often required in the calculation of force and torque, it is expected that the computation of the T-matrix pays off. Furthermore, as we will point out when we investigate the example cases, the T-matrix itself is of scientific value, as it contains all the information available concerning how an object interacts with light.

In the following section we outline the multipole decomposition of the fields [28]. This is the theoretical basis for the T-matrix calculation. The numerical calculation of the T-matrix is demonstrated in the next section. Finally, we provide four numerical examples in the form of interesting but simple objects the T-matrix of which we calculate to demonstrate the usefulness of the method.

## Multipole expansion of electromagnetic fields

We want do decompose the fields produced by scattering off of an illuminated object into contributions from different multipoles. We start by decomposing the total external fields surrounding the object into incident and scattered fields

[1]
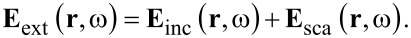


Note that this is always possible for theoretical considerations, but experimentally a strict distinction is not possible. In the following we largely skip the space and frequency dependency of the fields for simplicity but these dependencies are always assumed.

Then, the fields are expanded into vector spherical harmonic functions

[2]
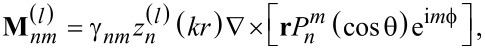


[3]
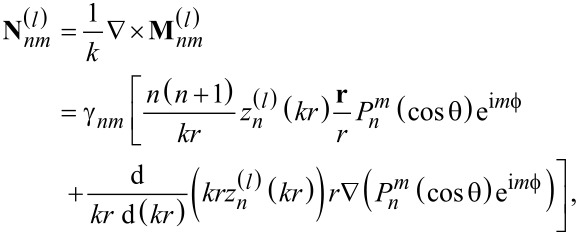


with the normalization factor

[4]
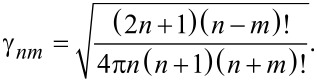


Here, the index *l* represents the underlying function: *l* = 1,2 corresponds to spherical Bessel functions of first *z^(1)^* = *j_n_* and second kind *z^(2)^* = *y_n_*, respectively, while *l* = 3,4 corresponds to Hankel functions of first and second kind 
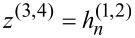
, respectively [29–30]. The incident field has to be finite in the center of the coordinate system. Thus, we have to take Bessel functions and the expansion reads as

[5]
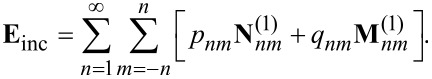


The scattered field needs to satisfy the outgoing Sommerfeld radiation condition and is expanded with Hankel functions

[6]
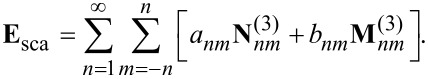


Here, *k* is the wave number in the surrounding medium, which is characterized by the permittivity ε(ω) and the permeability μ(ω). The complex expansion coefficients *a_nm_*(ω) and *b_nm_*(ω) are called scattering coefficients and the coefficients *p_nm_*(ω) and *q_nm_*(ω) are called incident coefficients. Together they contain all relevant information about the interaction of the particle with the given illumination. The physical meaning of the individual terms is also straightforward. For example in the expansion of the scattered field, the first term on the right hand side expresses the field scattered due to the induced electric multipole moments. The second term expresses the field scattered due to the induced magnetic multipole moments.

In order to be able to perform numerical calculations, we need to truncate the infinite sums in all expansions to a finite number *N*. This number is the multipole order that we take into account, *N* = 1 corresponding to dipoles, *N* = 2 to quadrupoles and so on. Thus, by choosing the maximum order we want to consider, we can strongly influence the computation time and final accuracy.

The scattering coefficients can be used to calculate the total scattering cross section of the system

[7]
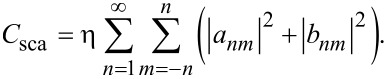


Here, η is a scaling factor, depending on the illumination. For a plane wave we have η = 1/(*k*^2^|**E**_0_|^2^), where **E**_0_ is the amplitude of the incident plane wave. In order to obtain more information, e.g., about a resonance, we can modify the equation to calculate the contributions of each multipolar order

[8]
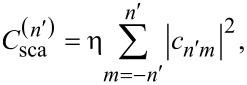


where *n*^′^ is the multipolar index and *c_nm_* stands for *a_nm_* if we want to consider the electric part or *b_nm_* if we consider the magnetic part, respectively. For example the electric and magnetic dipole contributions, which are usually the strongest contributions for electromagnetically small particles, read as

[9]
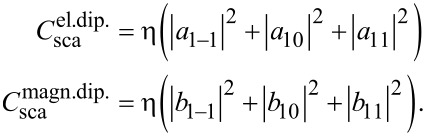


Thus, if we know the scattering coefficients of an object, we know exactly how the different multipoles contribute to the total scattering. That is particularly valuable if we want to design a specific multipole resonance of a nanoparticle or if we want to investigate a given resonance, e.g., from experimental measurements.

## T-matrix calculation

In the previous section we showed the decomposition of the fields into scattering and incident coefficients. Now we introduce a link between them that represents the interaction of the object with the illumination: the T-matrix

[10]
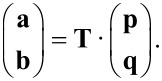


Here **a** and **b** are concatenated vectors that contain the scattering coefficients of the outgoing wave and **p** and **q** are concatenated vectors containing the coefficients of the incident field.

The T-matrix contains the entire near and far field information on how a specific object interacts with any illumination. It depends on the geometry and material composition of the structure in question. The T-matrix of a sphere is known analytically as the Mie coefficients. They form the T-matrix if they are ordered at the diagonal of the matrix. For arbitrary objects, however, the T-matrix is generally dense and is typically ordered in the following way

[11]
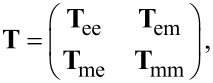


where **T**_ee_ describes coupling of electric incidence modes to electric scattered modes, **T**_mm_ describes the magnetic coupling and the other parts correspond to mixing of electric and magnetic modes. These parts of the T-matrix can be ordered to show the coupling between the multipolar modes

[12]
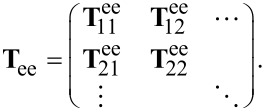


Then **T**nn' is the part that represents the coupling of order *n*′ to *n*. For a sphere, **T**_11_ would contain the first Mie coefficient *a*_1_ on the diagonal, **T**_22_ the second and so on.

Please note that the vector spherical harmonic functions 
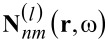
 and 
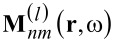
 in their presented form are normalized to the unit sphere. Therefore, also the T-matrix is normalized. In many cases it is crucial to use a normalized form of the T-matrix, e.g., when applying rotations [23,31] or for comparing the absolute value of different entries.

To perform simulations and to analyze a given object we need to calculate the T-matrix numerically. There are several established methods to calculate it, the first being the extended boundary condition method, originally introduced by Waterman [21,32–33]. Here, only homogeneous and isotropic particles can be considered and the method is quite time and resource consuming. A similar, more advanced possibility is the point matching method [34–35]. There, the coefficients of the incident, scattered and internal fields are related, but without the expensive surface integrations needed for the extended boundary condition method. Another established strategy to compute the T-matrix of an arbitrary object is to excite it with pure vector spherical harmonic functions to extract exactly one line of the T-matrix per illumination [22]. There, the fields are calculated with an integral equation solver. This method has the advantage that the incident field can be expressed in an exact way with a single incident coefficient. But the actual implementation of such an illumination with available tools is cumbersome. Similar methods have been proposed to retrieve the polarizability tensor of an object [36–39]. These techniques provide valuable information with comparatively little effort, but are restricted to dipolar objects.

Here, we propose a method that is easy to implement practically and, depending on the applied Maxwell solver, very efficient. We illuminate the particle with several different plane waves and extract the expansion coefficients by projecting the scattered fields onto vector spherical harmonic functions. To get the scattered field of the object for every plane wave, however, we need an additional tool that solves Maxwell’s equations for an arbitrary particle under plane wave illumination. Such tools are widely available. We chose here the finite element solver JCMsuite [12] because it is especially fast for the simulation of several different illuminations of an object at the same frequency. But in general, any full-wave solver that provides the scattered fields of an object could be used.

The scattering coefficients can be obtained from the scattered fields by Equation 13 and similarly the incident coefficients by Equation 14.

[13]
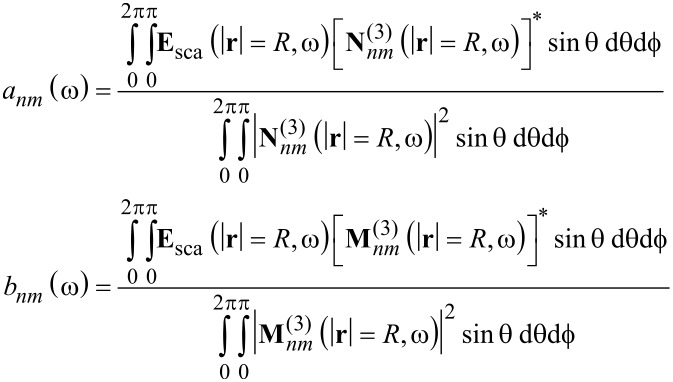


[14]
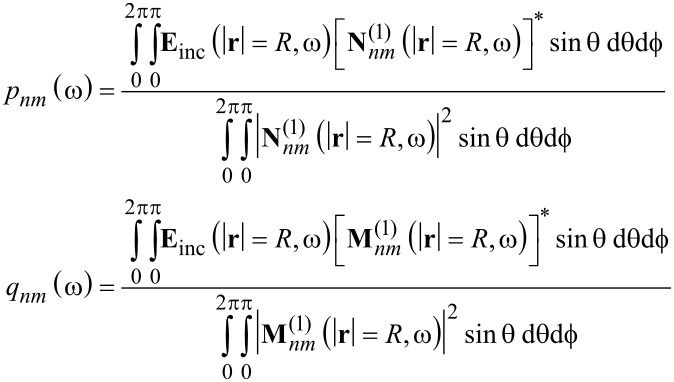


The fields are evaluated at a virtual sphere of fixed radius *R* that completely encloses the object. Analytically, the chosen radius has no influence on the result. Yet, in numerical realizations the choice may effect the result due to the finite sampling. It is advisable to use a small *R*, to keep the computational domain small, without touching the actual object. Note that we need to take the full details of the scattered field into account.

Now we get a set of 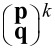 and corresponding 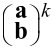 for each illumination *k*. Then we can construct a system of equations to calculate the numerical T-matrix **T***_K_* from Equation 10

[15]
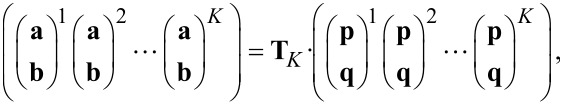


which we invert to find the T-matrix. In order to get an invertible system, we need to calculate the response from *K* illuminations, where *K* is equal to or greater than the rank *d* of the T-matrix. This rank depends on the multipole order *N* we want to take into account

[16]
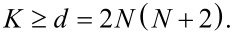


Using more illuminations is not strictly necessary, but will improve the accuracy of the result, as we will see in the following examples. The inversion of the rectangular matrix can then be done for example with QR decomposition [40]. Note that the resulting T-matrix is always a square matrix in our formalism.

## Results and Discussion

### Single sphere

As a first check to prove that the method delivers correct results, we investigate the most simple object: a single isolated sphere. We have the correct analytical solution for a single sphere available. The T-matrix has only the known Mie-coefficients on the diagonal and all other entries are zero.

Let us consider a single dielectric sphere with a radius of 100 nm and a relative permittivity of 16 in vacuum. The Mie-coefficients of this sphere for the first two orders are non-negligible at 600 THz. Such a high-permittivity sphere is nowadays at the focus of interest since it sustains a notable electric and magnetic dipole moment in the visible-light range.

**Figure 1:**
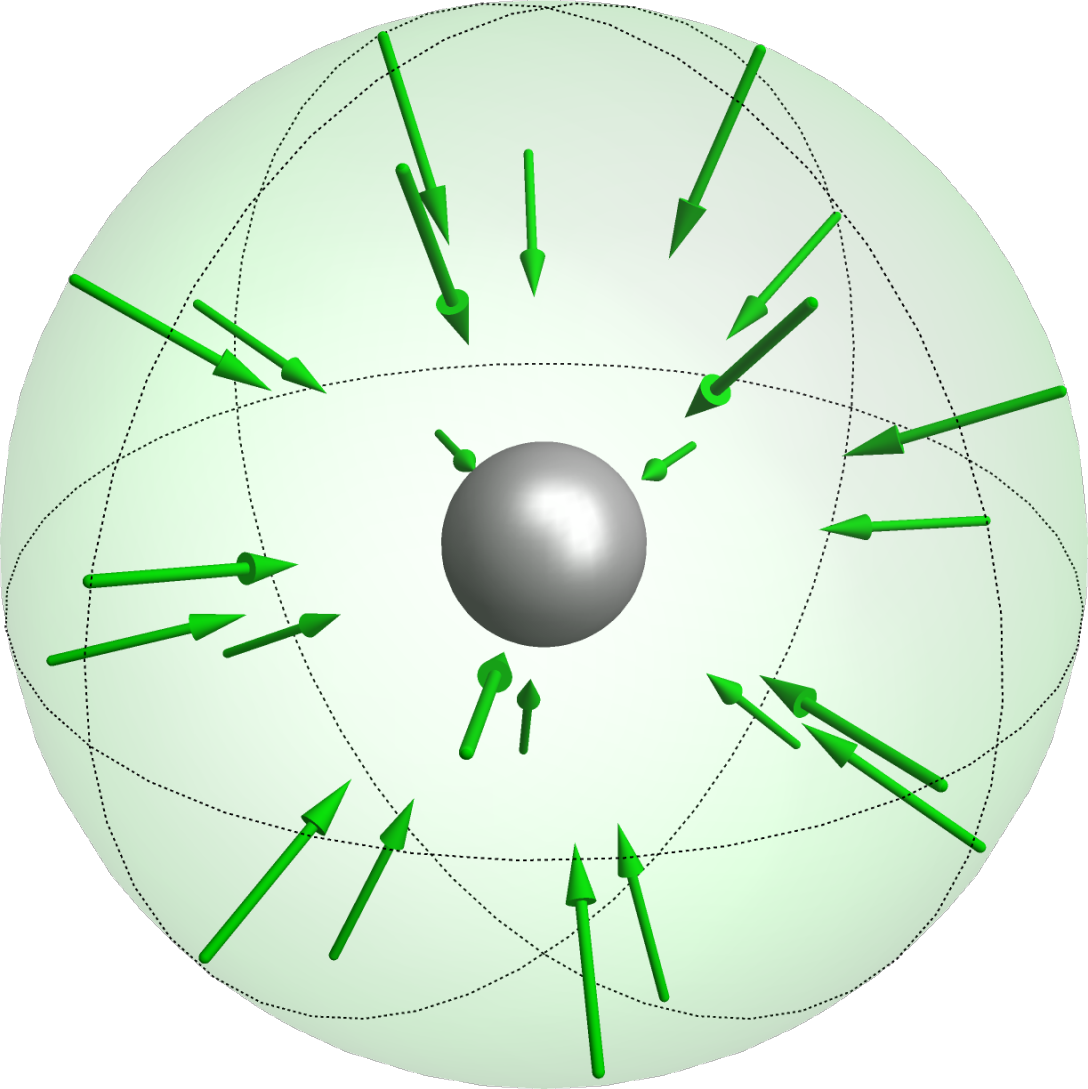
Single sphere with multiple plane wave illuminations, depicted with their wave vectors in green.

To calculate the T-matrix numerically we illuminate the sphere with the appropriate number of plane waves *K*. The wave vectors are chosen, such that they are evenly distributed in all directions. They can be considered as normal vectors of the surface of an imaginary sphere. They are distributed with the same nearest neighbor distance, as is shown in Figure 1. The polarization of the electric field is then chosen randomly for each wave.

To check the convergence of our method we increase the number of illuminations used to calculate the T-matrix. We introduce

**Figure 2:**
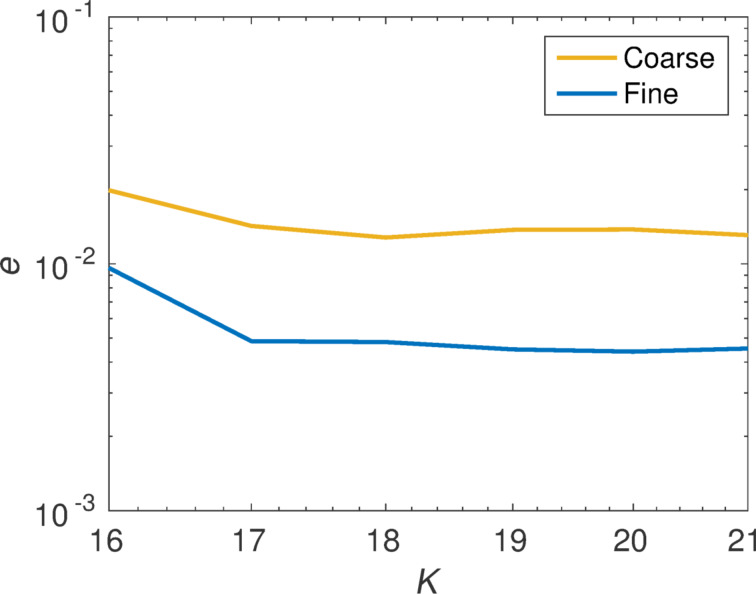
Convergence of the T-matrix calculation with the increase of the number of plane wave illuminations. The minimum number of illuminations to solve for all the unknown entries of the T-matrix for the multipole order *N* = 2 is 16. The error is shown for two different calculations, where the fine one has two times finer meshing.

[17]
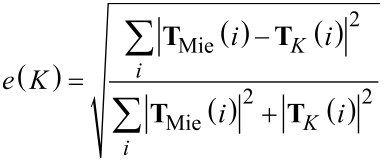


as a measure for the deviation of the T-matrix. Here, *(i)* represents an index that runs over all matrix components and **T**_Mie_ the analytically known T-matrix. This quantity is visualized in Figure 2 in a double logarithmic plot.

We clearly see that the coefficients can be reproduced with sufficient precision. The deviation drops at first if we increase the number of illuminations, then a plateau is reached. The remaining deviation can be explained by numerical errors introduced by the finite meshing of the sphere in the Maxwell solver as can be seen by considering a second calculation with a finer mesh.

### Dimer

We now consider a slightly more complex object to further investigate the convergence of our method. The dimer, i.e., two coupled spheres, is a suitable example, because we can calculate the T-matrix semi-analytically as a reference. We get the T-matrix of the single spheres directly from the Mie coefficients and construct the total T-matrix by translating the coefficients with addition theorems [41]. Please note that this solution is not completely accurate, because we truncate the infinite sums at *N*. Additionally, we calculate the T-matrix from the illumination with plane waves with a finite element solver, according to Equaiton Equation 15 and compare the results.

The object we investigate consists of two strongly coupled silver spheres with radius *r* = 30 nm and a center-to-center distance of *d* = 63 nm embedded in glass with ε = 2.25 [42]. We take established experimental data for the dispersive permittivity of silver [43]. Such objects can be fabricated in large quantities by self assembly methods, e.g., by connecting commercially available metal nanospheres with a linker molecule [44].

We set *N* = 2, because the higher orders do not contribute notably. For general objects we can construct a condition to find the optimal multipole order. We perform the T-matrix calculation with increasing multipolar order and check the contribution of each order by summing the corresponding T-matrix entries


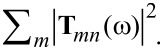


If we reach an order that has only a negligible contribution compared to the dominant ones, we stop the procedure.

**Figure 3:**
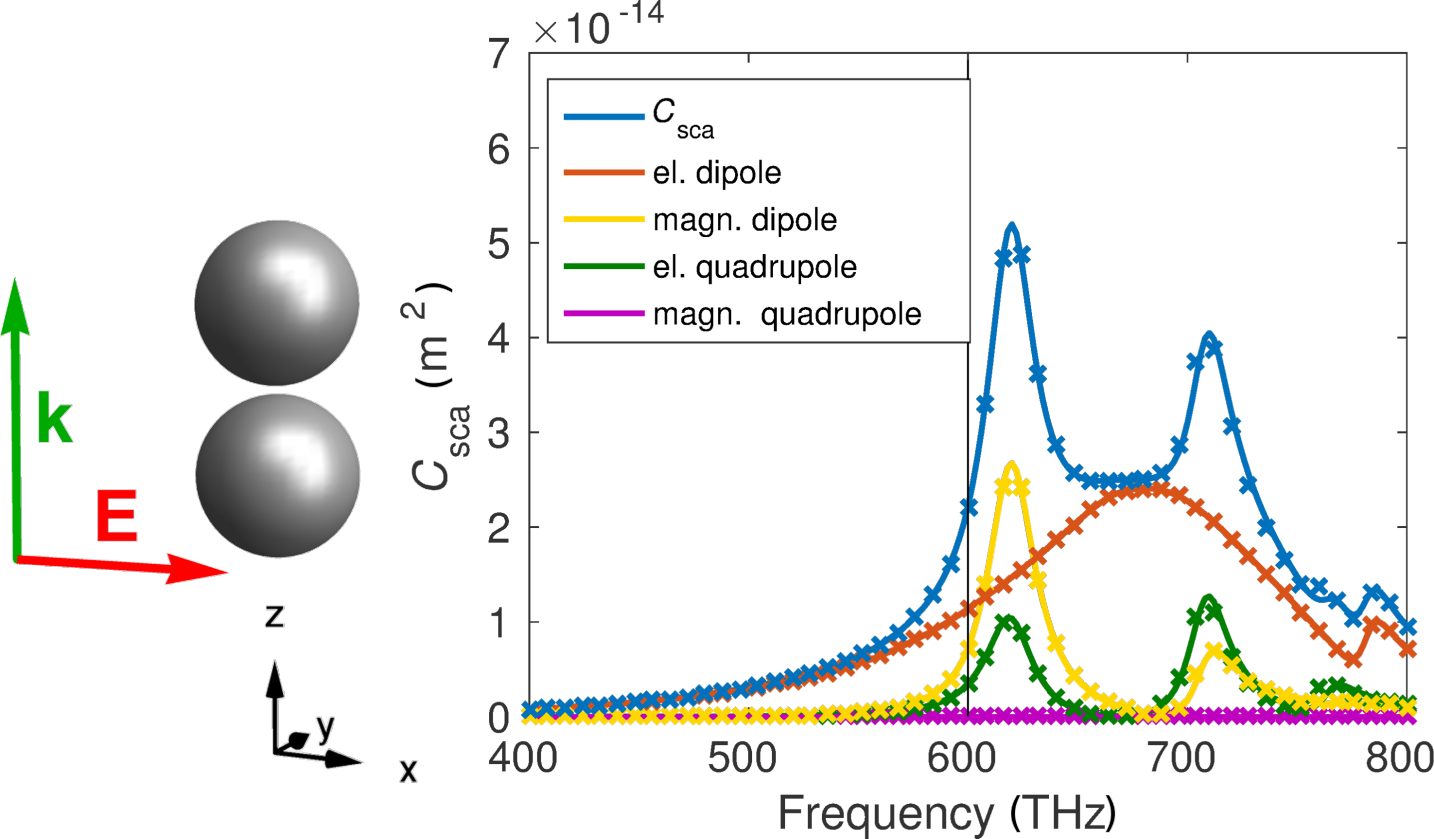
Total scattering cross section (blue) of the dimer and the contributions of different multipoles. The solid lines correspond to the semi-analytical calculation based on the Mie coefficients of the spheres and the crosses correspond to the calculation with the FEM solver. We observe an excellent visual agreement. The black vertical line shows the spectral position that is used for further calculations to show the T-matrix entries. 10 additional plane wave illuminations were used for the numerical T-matrix calculation. On the left side we display the geometry and illumination direction of the dimer setup. Note that this illumination is only relevant for this particular cross section calculation and the T-matrix is generally independent of the illumination.

As depicted in Figure 3, the scattering cross sections obtained from the different T-matrix methods agree very well. We can clearly identify three different resonances: a broad electric dipole resonance at 680 THz, and two resonances with magnetic dipole and electric quadrupole contributions at 620 and 710 THz, respectively. While the broad resonance is connected to the eigenmode of the single sphere, the two sharper peaks can be explained by a hybridization caused by the coupling of the two spheres and coupling of higher order multipole modes, respectively [42]. Here the multipole contributions are calculated according to Equation 8.

**Figure 4:**
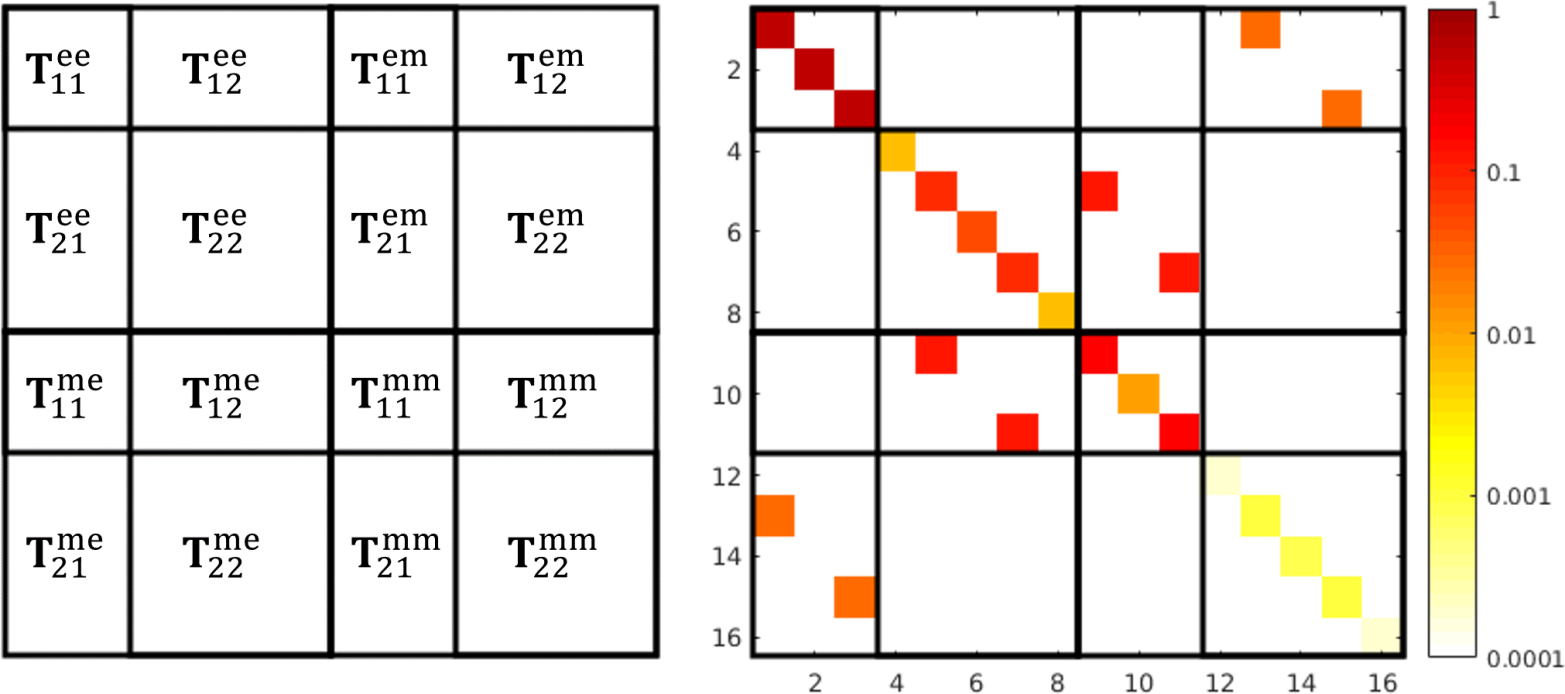
The left picture shows the general form of the T-matrix, as described in the previous section. For example 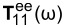 represents the electric dipole coupling. In the following we always present the T-matrix in this way. On the right hand side we see the absolute values of the T-matrix entries for the dimer on a logarithmic scale at 600 THz. This matrix was calculated semi-analytically with the Mie coefficients of the single spheres. The numbers on the axis count the rows and columns, respectively.

We get additional insights by looking at the entries of the T-matrix in Figure 4 at a discrete frequency. The way the T-matrix is presented in that figure will be the same in all the following figures showing the T-matrix. Note that the entries are complex numbers and we always display the absolute values. At first we notice that the dominant entries are connected to the electric dipole 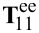 and, to a lesser extent, to the magnetic dipole 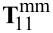 and electric quadrupole components 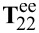. Additionally, we see the aforementioned cross coupling between magnetic dipole and electric quadrupole in the submatrix 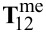 and all permutations. This cross coupling also became evident in the scattering cross section.

**Figure 5:**
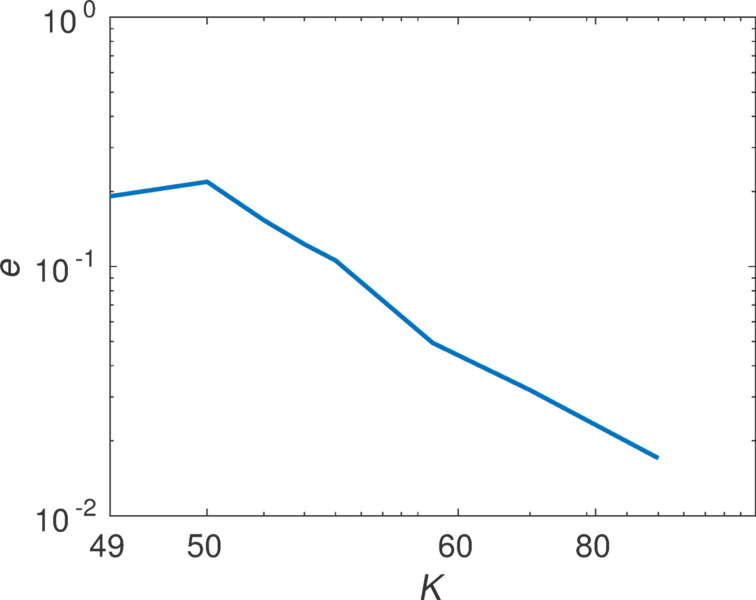
Convergence of the T-matrix calculation with the increase of the number of plane wave illuminations. The minimum number of illuminations necessary to solve for all the unknown entries of the T-matrix for the multipole order *N* = 4 is 48.

**Figure 6:**
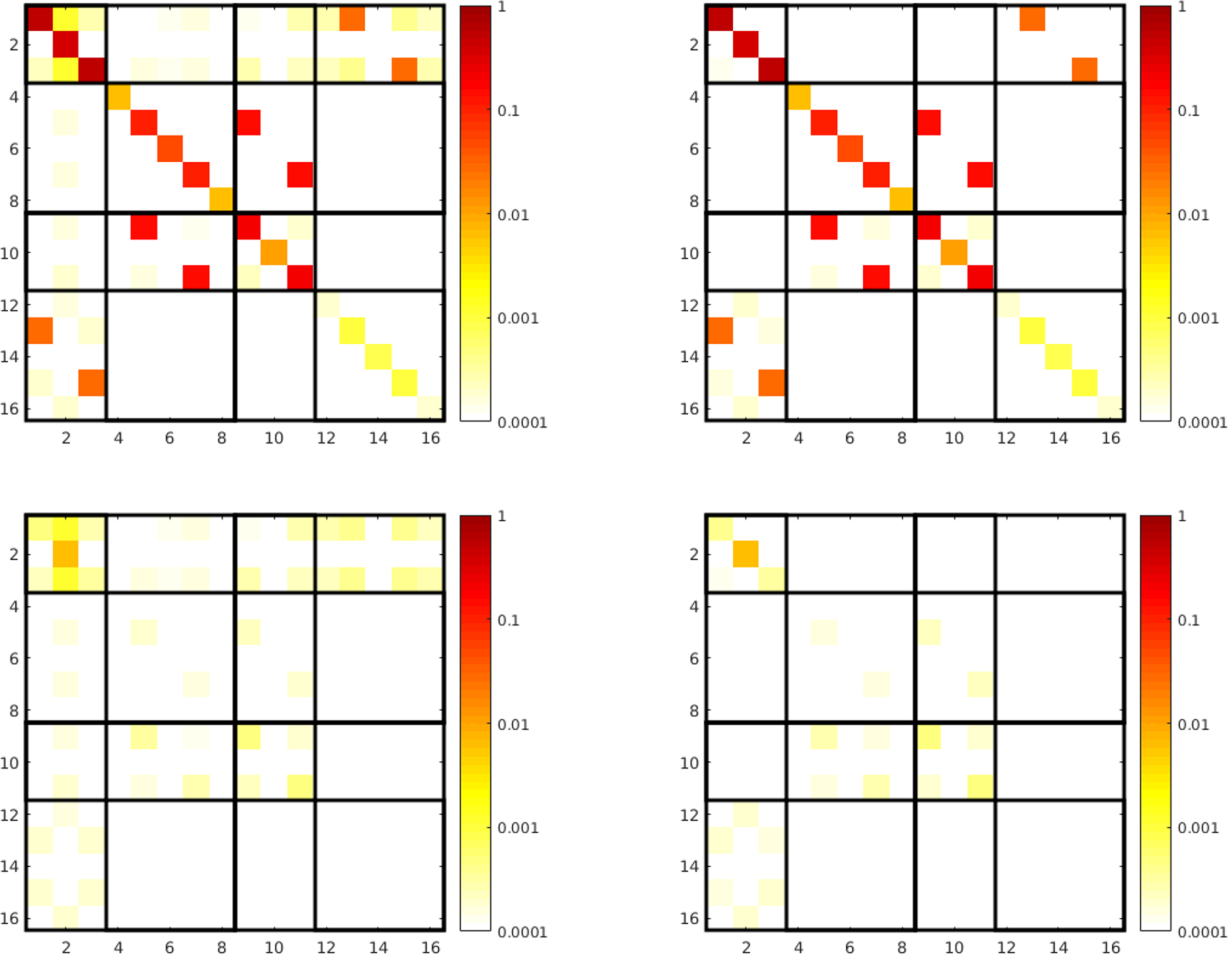
T-matrix entries of the dimer, calculated with the presented method at 600 THz. For the sake of clarity we show only the parts connected to the lowest two multipole contributions. On the left hand side we see the matrix with the minimum number of plane wave illuminations 48, on the right hand side with 50 extra illuminations. The bottom row shows the absolute difference to the semi-analytic solution 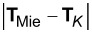.

Now, we investigate the behavior when we increase the number of illuminations *K* in the T-matrix calculation. We consider the T-matrix with *N* = 4 at the frequency 600 THz, where several multipole orders contribute to the total scattering. As a reference, we take the calculation based on Mie coefficients. In Figure 5 we can observe the expected behavior of the error, the deviation goes down exponentially as we increase the number of plane wave illuminations. So the more complicated structure actually benefits from multiple additional illuminations. In Figure 6 we see the T-matrix entries of two different numbers of illuminations. We clearly see that the numerical noise, i.e., the parts of the matrix that should be zero, is going down significantly.

In conclusion, we can state that the presented method delivers correct results and that it is highly beneficial to use a few illuminations more than absolutely necessary.

### Sandwich particle

Another interesting and in a sense similar object is the sandwich particle. It consists of two metallic disks, usually separated by a dielectric spacer layer. This particle is preferably fabricated by top-down procedures, such as electron beam lithography [5,45].

**Figure 7:**
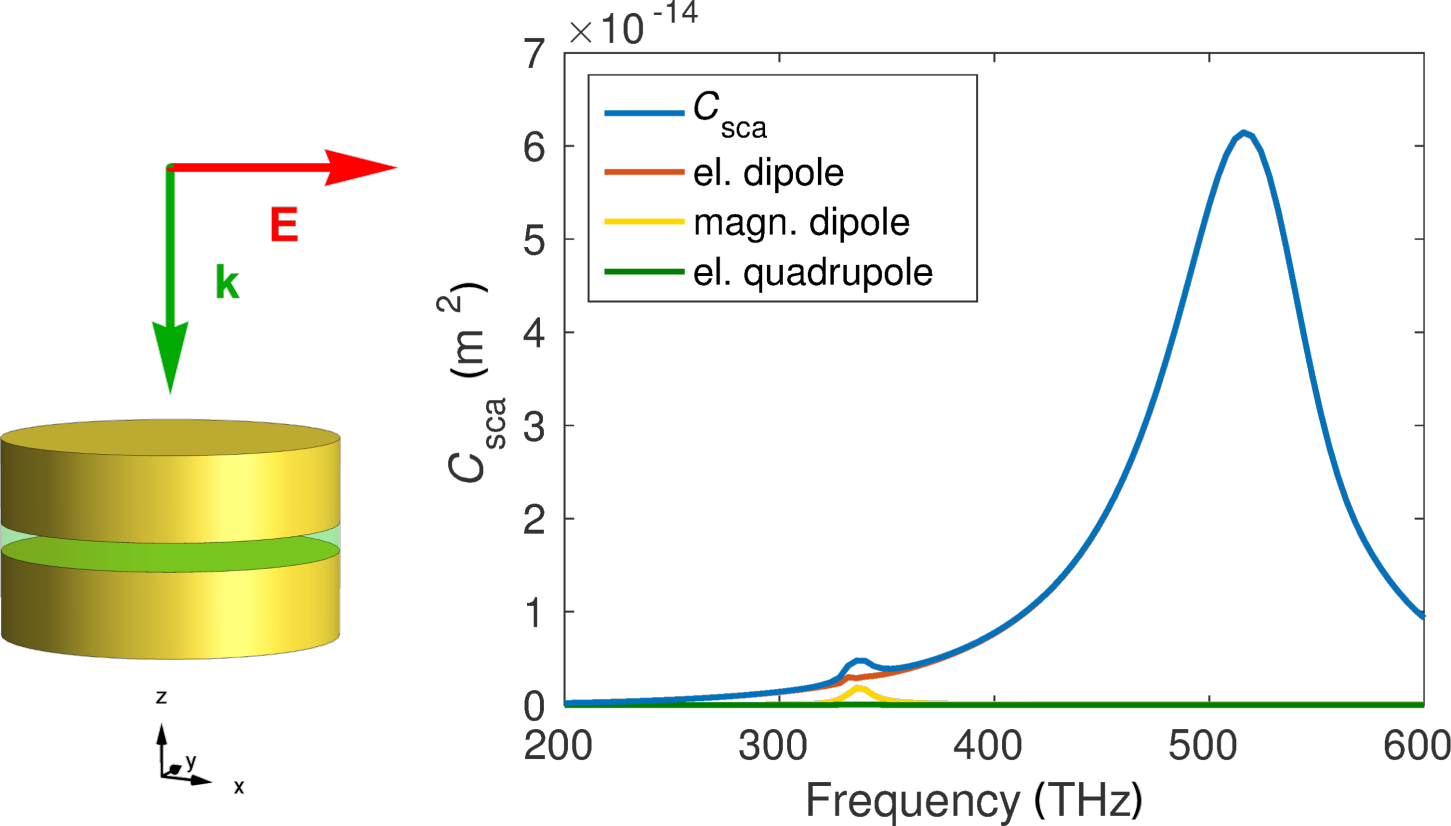
Total scattering cross section (blue) of the sandwich particle and the contributions of different multipole orders. We observe an electric and a magnetic dipole resonance. On the left side we display the geometry and illumination direction of the sandwich. Note that this illumination is only relevant for this particular cross section calculation and the T-matrix is generally independent of the illumination.

The investigated object has a disk radius of 60 nm, a disk height of 30 nm and a gap thickness of 10 nm. The metal disks consist of gold [43] and the spacer is made of SiO_2_ where we assumed a constant permittivity of ε = 2.13 and the surrounding is vacuum. Similarly to the dimer case, the two separated disks support a magnetic dipole resonance at 336 THz, as can be seen in Figure 7. Additionally, there is a strong and broad electric dipole resonance at 520 THz that is, again, attributed to the resonance of a single disk.

We can observe a magnetic resonance that is weaker than in the case of the dimer, but the big advantage is that the peak is well isolated from other resonances. Furthermore, we observe no higher multipole-order contributions at all in the investigated frequency region. This is particularly beneficial for homogenization, because we can apply the Clausius–Mosotti relation, which is only valid in dipole approximation. The different geometry makes it now possible to shift the electric and magnetic dipole resonances almost independently, because we have more parameters. The electric mode depends strongly on the radius of the disks, while the magnetic mode can be shifted by changing the thickness of the gold and spacer layers, respectively.

**Figure 8:**
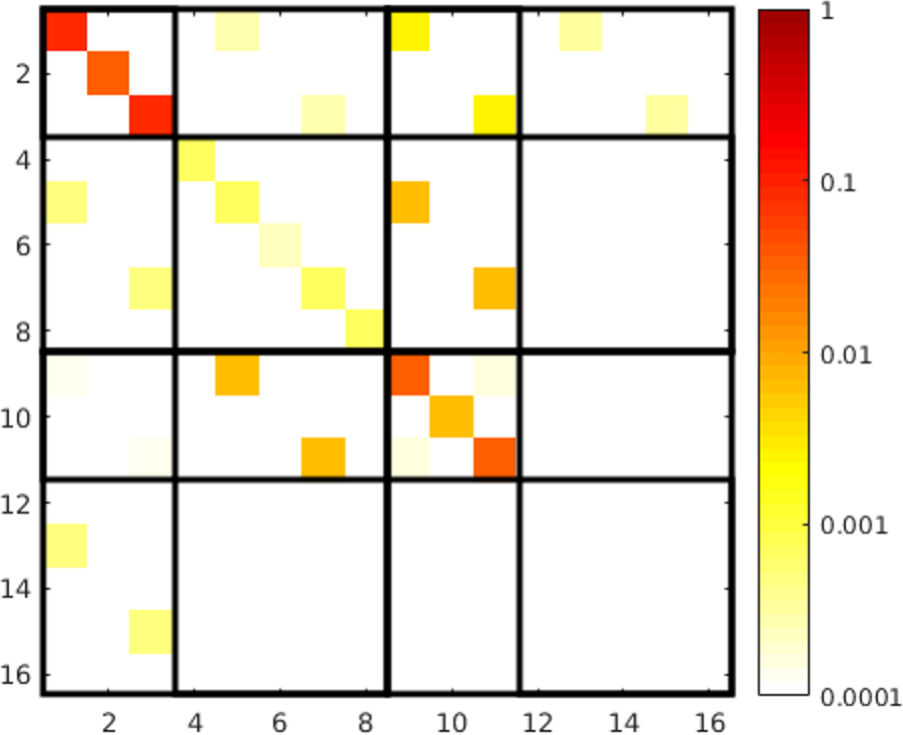
T-matrix entries of the sandwich particle, calculated with the presented method at 336 THz with 50 extra illuminations. See Figure 4 for a description of the submatrices.

Again, we can get a deeper understanding of the response, by looking at the T-matrix. Figure 8 shows the entries of the matrix of the presented structure at the position of the magnetic resonance, 336 THz. We notice a strong similarity to the previous case of the dimer. This is because the object has almost exactly the same geometrical symmetries. However, we also see some notable differences. Firstly, the particle can be described in very good approximation as a dipole, because the parts corresponding to higher multipoles are negligible. Furthermore, the cross coupling between the magnetic dipole and the electric quadrupole is significantly lower than in the previous case. This is the cause of the well isolated magnetic resonance we saw in the scattering cross section in Figure 7 in contrast to the mixed magnetic dipole and electric quadrupole resonances of the dimer.

Additionally, we can deduce the preferential orientation of the induced multipoles from the pattern of the T-matrix. Let us investigate the electric dipolar part and call the entries

[18]
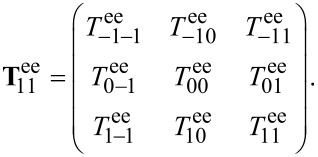


Now 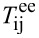 connects the incident coefficient *p*_1j_ to the scattering coefficient *a*_1i_. The same is true for all the other parts of the T-matrix, with adjustments for the higher-order parts. The entry 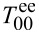 is special because it is connected to the *z*-axis. The *z*-axis is distinguished in the definition of the vector spherical harmonic functions. This means that if this entry has a large absolute value, the corresponding object will exhibit a strong electric dipole in *z*-direction, 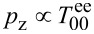. Conversely the dipole moments in the *x*–*y*-plane are proportional to linear combinations of entries with ±1 indices. This also translates directly to the magnetic part of the T-matrix. Considering this, we investigate again Figure 8. We see that the electric dipole is almost isotropic. This becomes clear when we compare the T-matrix to that of a sphere, as stated above, we would get 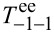 = 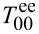 = 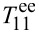 in this completely isotropic case. The magnetic dipole, however, is much stronger in the *x*–*y* plane because of the geometry of the investigated object with the gap. The magnetic dipole moment is induced by antiparallel currents in the metal disks. Please note that these considerations are completely independent of the illumination, because we investigate the T-matrix directly.

### Helix

Now we can utilize the biggest advantage of the proposed method, namely that we can calculate the T-matrix of an arbitrarily complex object. To showcase this we compute the T-matrix of a metal helix. In this case we can not make use of inherent symmetries of the object a priori and have no analytic solution available.

**Figure 9:**
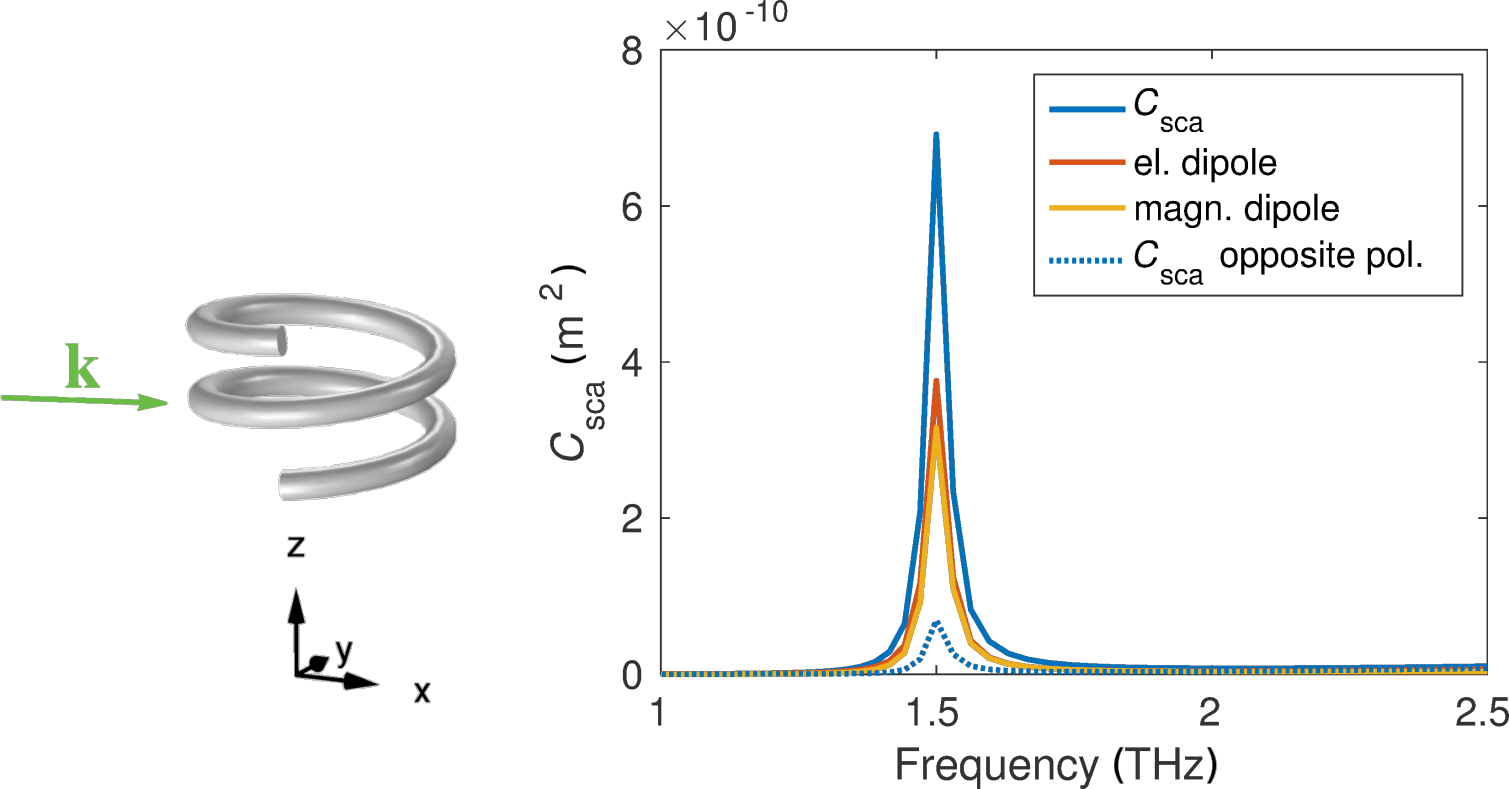
Right-handed metallic helix with two windings. Total scattering cross section for right-handed (solid) and left-handed (dotted) circular polarization. The electric and magnetic dipole contributions are approximately equal. On the left side we display the geometry and illumination direction of the helix.

Calculating the T-matrix is particularly interesting for the present object, because numerous electromagnetic properties can be deduced from its entries, such as the duality conservation and electromagnetic chirality [46]. The helix was optimized to express a strong electromagnetic chirality at a specific frequency and, thus, shows a strong contrast in the scattering cross section for two opposite circular polarized incident fields. The major radius of the helix is 6.48 μm, the pitch is 8.52 μm, the minor radius is 0.8 μm, and the helix is surrounded by vacuum. The operating frequency of the system, i.e., the position of the resonance is at 1.5 THz. As we see in Figure 9, there is a strong difference in the scattering cross section if we illuminate with right-handed or left-handed circularly polarized light.

For the following considerations it is beneficial to introduce shortly the concept of duality symmetry. In free space, Maxwell’s equations are invariant under the transformation [47]

[19]
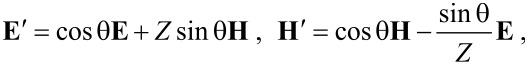


where **E** is the electric field, **H** is the magnetic field, *Z* is the impedance and θ is an arbitrary constant angle. The symmetry that is corresponding to this transformation invariance is duality. Helicity is defined as the product of the total angular momentum **J** and the direction of the linear momentum of the wave **P**/|**P**|[48]

[20]
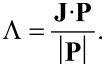


It has the two eigenvalues 1 and −1, and the corresponding eigenstates [49]

[21]
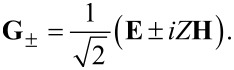


For example, a circularly polarized plane wave is a state of pure helicity and it is preserved if duality symmetry applies.

We can see directly that the object is approximately dual at the resonance frequency of 1.5 THz, because the magnetic and electric dipole contributions are balanced and no higher multipole contributions arise [50]. This means that the interaction of the particle with the incident light does not change the helicity of the incident waves.

**Figure 10:**
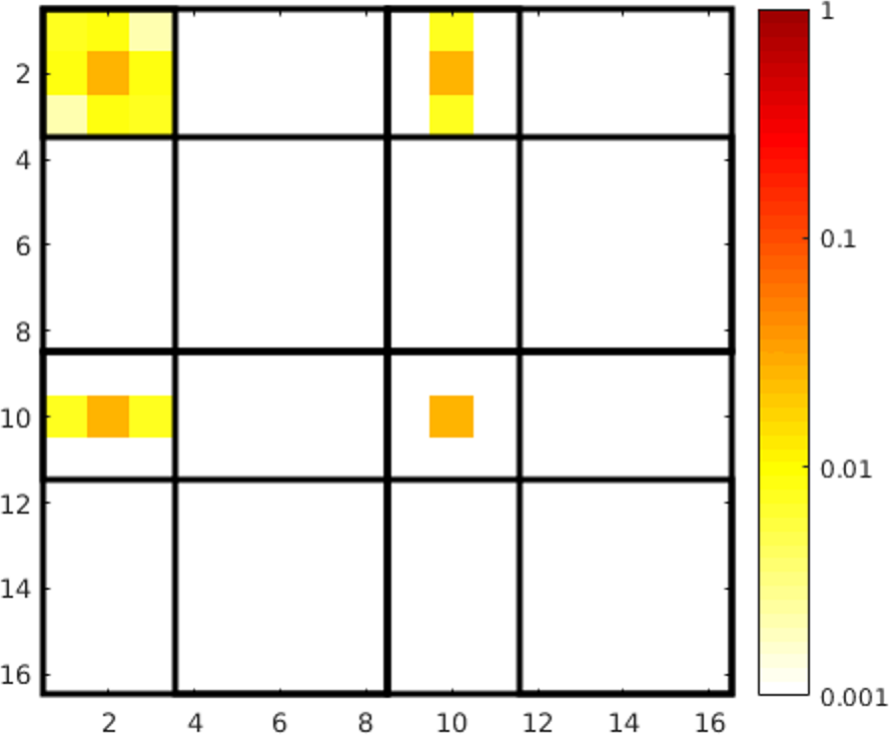
T-matrix entries of the right handed helix, calculated with the presented method at 1.5 THz with 10 extra illuminations. See Figure 4 for a description of the submatrices.

Looking at the T-matrix entries in Figure 10 we see, like in the previous cases, that the object can be described mainly by the dipole contributions. The interesting aspect here is that the electric and magnetic dipoles have almost the same strength and are both aligned along the *z*-axis at the same frequency. Furthermore the mixing of electric and magnetic dipole moments is also very strong. This means that an electric dipole is induced by a magnetic dipole excitation and vice versa. All these properties lead to the approximate duality symmetry of the object.

As stated above, we can calculate measures for the duality breaking, electromagnetic chirality and the scattering cross section contrast for general plane wave illuminations from the T-matrix entries. This is done by transforming the T-matrix from its current form to the helicity basis

[22]
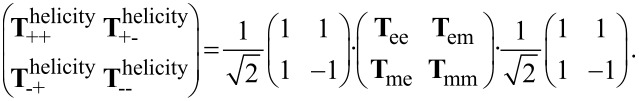


**Figure 11:**
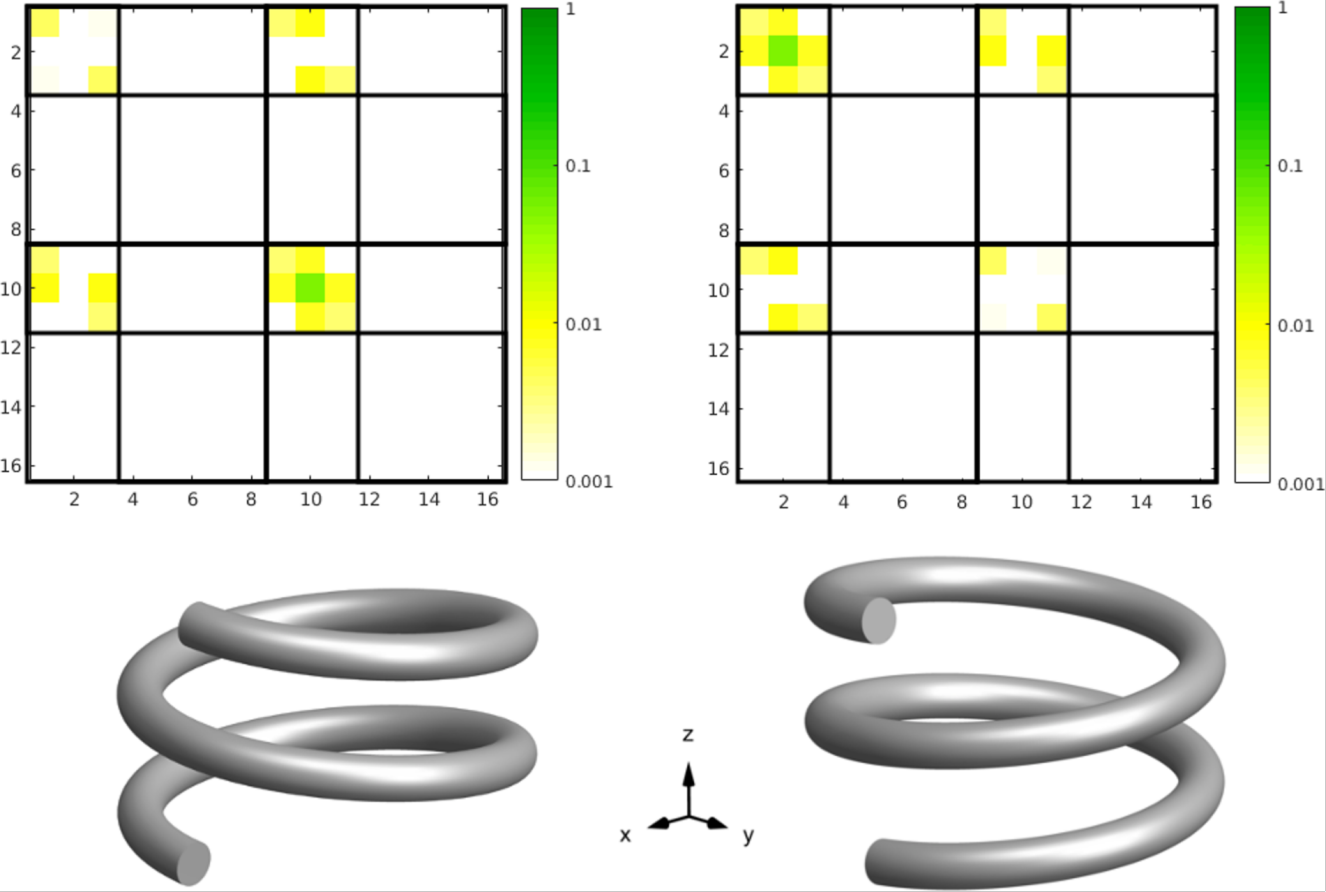
T-matrix entries in the helicity basis (obtained by Equation 22) of a left-handed and a right-handed helix. We clearly observe that each helix interacts primarily only with one helicity and that the mixing of different helicities is very low (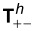 and 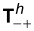). The T-matrices are ordered, in principle, in the same way as shown in Figure 4, but with helicity + and − states instead of e and m.

The submatrices represent now the coupling of the helicity + and − states. In Figure 11 the entries of the transformed matrix are depicted for a left-handed and a right-handed helix. We can clearly distinguish the two cases, because each helix interacts mainly only with one helicity. The left handed helix interacts with helicity − (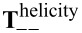) states and the right handed interacts with helicity + (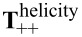).

To further analyze the T-matrix of the object we can perform a singular value decomposition (svd) of the submatrices [46]. The values are ordered to get the vectors


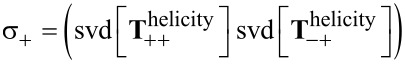


and


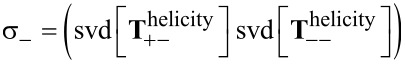


from which we calculate the quantities that are related to helicity

[23]
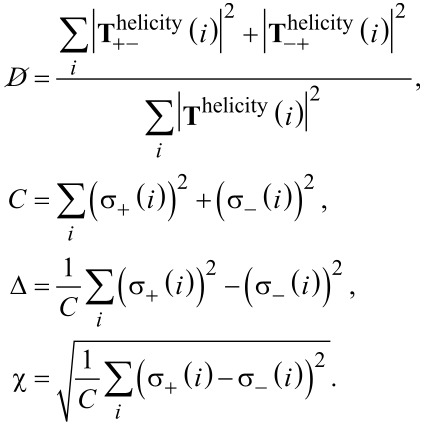


**Figure 12:**
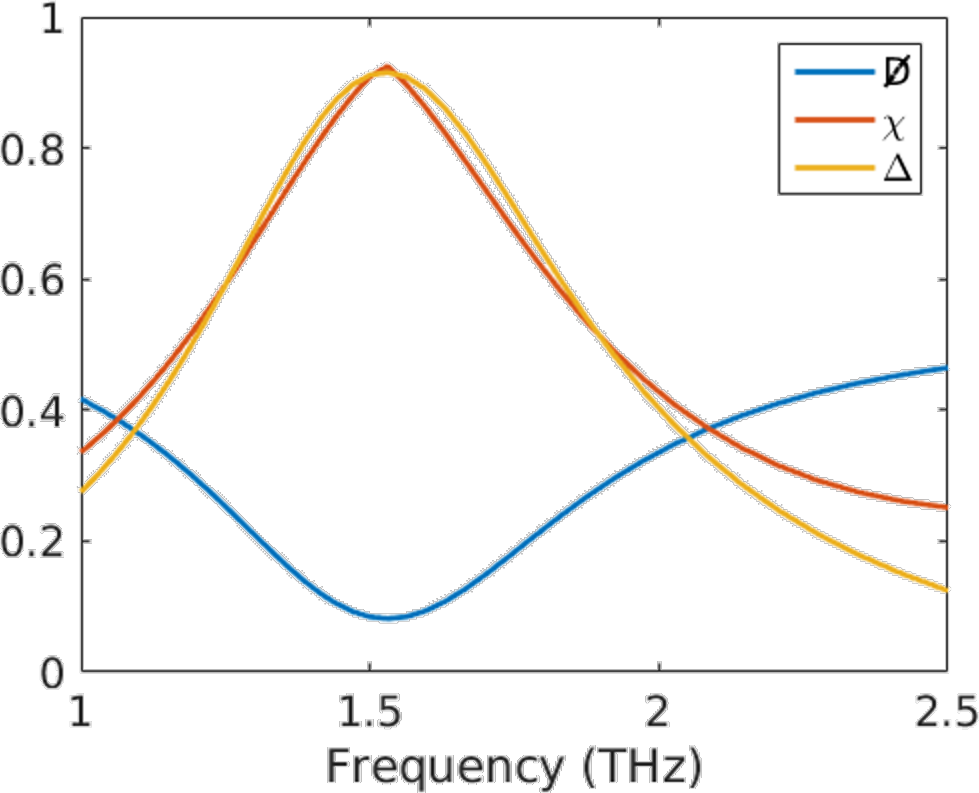
Duality breaking, electromagnetic chirality and cross section contrast of the left-handed helix.

Here, 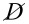 is the duality breaking, *C* can be interpreted as the averaged total scattering cross section independent of a specific illumination, Δ is the cross section contrast for different circular polarizations, and χ is a measure for the electromagnetic chirality [46]. The values for the chosen helix are shown in Figure 12.

We clearly see the anticipated minimum in the duality breaking. This means that the helix is approximately dual at the resonance frequency. Simultaneously the chirality reaches a maximum of 0.9, which means that the object responds differently to the two opposite polarizations of illuminating radiation. This is also evident from the strong cross section contrast.

The presented quantities, and possibly others, are only accessible because we calculated the T-matrix of the object. This information makes it also possible to optimize such objects numerically to achieve the desired behavior.

## Conclusion

We demonstrated an algorithm to calculate the T-matrix of an arbitrary object. We showcased numerical examples by calculating the T-matrix of four objects. Especially the calculation of the silver helix demonstrates the strength of the approach. We investigated properties that are usually not available with established full wave solvers.

The T-matrix is calculated with the help of widely available and well established tools to calculate the scattered fields upon plane wave illumination. The T-matrix contains the electromagnetic scattering information independently of the illumination. By investigating the matrix, we get valuable information about the multipolar composition of the scattering cross section and electromagnetic chirality. This is extremely useful for understanding the nature of a given response.

Furthermore, the T-matrix can be used in an extended multiscattering formalism to calculate the response of a whole cluster of objects efficiently. This will speed up calculations by a considerable amount.
