# Correction: Computing the T-matrix of a scattering object with multiple plane wave illuminations

**DOI:** 10.3762/bjnano.9.88

**Published:** 2018-03-22

**Authors:** Martin Fruhnert, Ivan Fernandez-Corbaton, Vassilios Yannopapas, Carsten Rockstuhl

**Affiliations:** 1 Institute of Theoretical Solid State Physics, Karlsruhe Institute of Technology, Wolfgang-Gaede-Strasse 1, 76131 Karlsruhe, Germany; 2 Institute of Nanotechnology, Karlsruhe Institute of Technology, P.O. Box 3640, 76021 Karlsruhe, Germany; 3 Department of Physics, National Technical University of Athens, Heroon Polytechniou 9, 15780 Zografou, Greece

**Keywords:** metamaterials, nanooptics, numerics, scattering, T-matrix

In the original publication, the unnumbered equation that appears in the top right corner on page 624 before Equation 23 contains an error. The order of the svd vectors should be as follows:


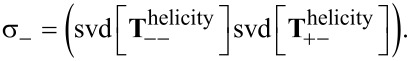


The authors would like to acknowledge Mr. Philipp Gutsche for pointing out this error.

